# Evidence for the effectiveness of anti-hypertensive medicines included on the Chinese National Reimbursement Drug List

**DOI:** 10.1186/s12913-019-3937-0

**Published:** 2019-02-11

**Authors:** Wenbin Liu, Lizheng Shi, Monika Sawhney, Xiaoli Gu, Yingyao Chen

**Affiliations:** 1 Department of Health Management, School of Public Health, Fujian Medical University, Fuzhou, Fujian China; 2 School of Public Health and Tropical Medicine, Tulane University, New Orleans, LA USA; 3 Department of Public Health Sciences, College of Health and Human Services, University of North Carolina at Charlotte, Charlotte, NC USA; 4 Department of Discipline Inspection, Children’s Hospital, Nanjing Medical University, Nanjing, Jiangsu China; 5 Key Lab of Health Technology Assessment (Ministry of Health), Collaborative Innovation Center of Social Risks Governance in Health, School of Public Health, Fudan University, 446 Zhaojiabang Road Building 2 Room 1001, Xuhui District, Shanghai, 200032 China

**Keywords:** Evidence support, National Reimbursement Drug List (NRDL), Antihypertensive medicines, Urban employee basic medical insurance (UEBMI), China

## Abstract

**Background:**

Evidence-based decision on drug list or formulary has been applied worldwide. Although the importance of scientific evidence was emphasized, the decision-making procedures for including medicines into the national reimbursement drug list were often challenged by their process opacity and relying on subjective expert opinion. This study aimed to explore and assess the evidence for the effectiveness of anti-hypertensive medicines included on the Chinese National Reimbursement Drug List (NRDL), and to provide recommendations for further improvement.

**Methods:**

Three international evidence-based guidelines were selected to serve as reference criteria. The antihypertensive medicines included in NRDL of Urban Employee Basic Medical Insurance (UEBMI) were compared with recommended drugs in three international guidelines. Medicines recommended by at least two guidelines were considered to have sound evidence support for the effectiveness. Otherwise, published literature with high evidence grade, namely systematic review, meta-analysis and randomized controlled trial (RCT), were searched for further assessment. Medicines reported as fairly good effectiveness by literature with high evidence grade can be also considered having sound evidence for the effectiveness. Methodological quality of systematic review or meta-analysis was evaluated by AMSTAR scale and PRISMA statement. Literature quality of RCTs was assessed by Jadad scale.

**Results:**

For the 97 antihypertensive medicines in NRDL, there were sound evidence supports for the effectiveness of 56 kinds of medicines. Specifically, twenty-six of them were supported by international evidence-based guidelines, twenty were supported by systematic review or meta-analysis and the other ten by RCT. However, for the rest 41 medicines, there is insufficient evidence for their effectiveness.

**Conclusions:**

Some antihypertensive medicines in NRDL did not have sufficient evidence for their effectiveness. Further evaluation and revision were required. It is also recommended to standardize decision-making procedures for inclusion of medicines, set up high quality evidence database to timely provide sound evidence, and so on.

**Electronic supplementary material:**

The online version of this article (10.1186/s12913-019-3937-0) contains supplementary material, which is available to authorized users.

## Background

Evidence-based decision on drug list or formulary has been applied worldwide [1]. After proposing the recommendation that using evidence-based principles in the selection of essential medicines, World Health Organization (WHO) introduced the methods of Grading of Recommendations Assessment, Development and Evaluation (GRADE) into the document “WHO Handbook for Guideline Development- 2003”, which comprehensively take research design, quality and grade of evidences into consideration [2]. In 2009, WHO explicitly recommended to apply GRADE in evidence-based evaluation and selection of essential medicines, as well as submit the evidence of medicine efficacy and safety in the form of GRADE tables [2, 3].

In China, National Reimbursement Drug List (NRDL) of Urban Employee Basic Medical Insurance (UEBMI) is an important guidance list for production, supply, use, supervision, and management of included medicines. The medicines in this list are categorized into Class A and Class B. The medicines in Class A are often widely used in clinical treatment and have good curative effect, while the medicines in Class B alternatively using in clinical practice also have well treatment efficacy, but higher price [4]. The NRDL of UEBMI was firstly issued by Ministry of Labor and Social Security (MLSS) in 2000. The list was adjusted approximately every five years to make sure the included medicines are safe, effective, economic, and fulfilling basic clinical needs. The provincial bureaus have no right in adjusting medicines in Class A, but they have the right to adjust not more than 15% of the medicines in Class B in accordance to local socio-economic level, health demand and medication habits [5].

Although the importance of scientific evidence was emphasized, which has also promoted the renewal and improvement in selection of medicines into the NRDL of UEBMI to some extent, the further improvement of the evidence-based evaluation system and working mechanism are still in urgent need for China [6, 7]. For many years, the list of alternative medicines to be included in NRDL of UEBMI was often formulated on the basis of national essential drug list and the need of certain medical services. To determine the medicines to be included in the updated NRDL, some experts were randomly selected from the MLSS expert database to vote. The medicines receiving more experts’ support are likely to be included [8]. However, this decision-making procedure relies too much on expert opinion, which are mainly based on subjective experience rather than objective evidence. It greatly increases the risk of including inappropriate medicines in the NRDL, which may directly lead to inefficient use of health insurance funds and great adversity to public health.

For the wide coverage of UEBMI medicines, it is of great importance to use evidence-based methods to ensure their safety, efficacy, and cost-effectiveness. In consideration of their public health significance, this study takes the antihypertensive medicines as example to examine the situation of evidence support for the effectiveness of the medicines in the NRDL of UEBMI. The findings can provide recommendations for further revision of NRDL and the improvement of evidence-based decision-making.

## Methods

To objectively judge whether the effectiveness of certain medicine was supported by sound evidence, we set a strategy by reviewing international evidence-based medicine lists in combination with published research paper of high evidence grade.

### Selection criteria of international evidence-based medicine lists

Scientific evidence support and representative are two prerequisites for the selection of international evidence-based clinical guidelines or medicine lists as reference criteria. Representativeness implies the generality to a certain extent, while the scientific evidence support means the development of certain guidelines or drug lists following a formal and scientific procedure, such as searching and screening available high-level evidence, extracting, synthesizing and assessing data by rigorous methods, and so on.

Comprehensively considering the two elements mentioned above, three international evidence-based clinical guidelines or medicine list were selected: the WHO Essential Medicine List of 2017 version (EML 2017) [9], the Eighth Report of the Joint National Committee on Prevention, Detection, Evaluation and Treatment of high blood pressure (JNC 8) [10], and the Clinical Management of Primary Hypertension in Adults (NICE 2011) [11].

### Literature search strategy

For each drug in the NRDL, a literature search was performed using the following databases: Pubmed, Medline, Web of science, China National Knowledge Infrastructure (CNKI), Wangfang Data, Chinese Bio-Medicine (CBM) database. The search started with broad search terms, such as the genetic name or commonly used brand name of certain medicine included in the current NRDL of UEBMI to treat hypertension, then narrowing down to the field of effectiveness with the time span between Jan 1st, 1995 and March 31st, 2017.

### Literature inclusion criteria

Clinical studies focusing on the certain medicines’ effectiveness of the anti-hypertension with the research design as meta-analysis, systematic review, or randomized controlled trials between Jan 1st, 1995 and March 31st, 2017.

### Literature exclusion criteria

1. Studies mainly focusing on economic or cost analysis
2. Studies that were not designed as meta-analysis, systematic review, or randomized controlled trials.
3. Meta-analyses or systematic reviews that were not the latest ones on the effectiveness of certain medicines.
4. Clinical studies not published in English or Chinese.

### Literature quality assessment

The literature quality of the searched articles were subsequently reviewed and scored by two independent reviewers under the guidance of the scales or checklists mentioned below. If there were differences in the scores between the two reviewers, they were further discussed for consensus purpose.

### Quality assessment for systematic review and meta-analysis

The reporting quality of systematic review and meta-analysis were assessed by using Preferred Reporting Items for Systematic Reviews and Meta-Analyses (PRISMA) statement. The checklist has 27 items about the literature integrity, such as objection in the introduction section, data collection process in the methods section, summary of evidence in the discussion section and so on [12]. If certain item was fully reported, partly reported, not reported at all, it will get 1 point, 0.5 point and 0 point, respectively. Therefore, the total score ranges from 0 to 27 points. Usually, systematic review or meta-analysis with PRISMA scoring more than 21 points can be considered fairly acceptable in literature integrity.

Meanwhile, the methodological quality of systematic review and meta-analysis were assessed by applying A Measurement Tool for the ‘Assessment of multiple systematic reviews’ (AMSTAR). The scale contains 11 items, such as “Was an “a priori” design provided?”, “Was a list of studies (included and excluded) provided?”, and so on. If the answer is “Yes”, “Partly” and “No”, it will get 1 point, 0.5 point and 0 point, respectively. Therefore, the total score ranges from 0 to 11 points [13]. By convention, the methodological quality of systematic review and meta-analysis with AMSTAR scoring more than 7 points can be considered acceptable.

### Quality assessment for RCT

The methodological qualities of the included RCT were assessed by Jadad scale, which contains three items, such as “Randomization”, “Double blinding” and “Withdrawals and dropouts” [14]. For the first 2 items, if certain item was “described and appropriate”, “only described”, “not mentioned”, it will get 2 points, 1 point and 0 point, respectively. For the last item “Withdrawals and dropouts”, if it was described, it will get 1 point, or else, 0 point. Therefore, the full mark of the adjusted Jadad scale is 5 points. Besides, the methodological quality of RCT with Jadad score not less than 3 points can be considered acceptable.

### Analysis strategy

There are three steps as follows to assess the evidence for the effectiveness of anti-hypertensive medicines included on the NRDL.

Step one, comparison among the drug lists. If certain medicine included in NRDL of UEBMI was also included in at least two international evidence-based clinical guidelines or medicine lists mentioned above, it can be perceived that the effectiveness of this medicine was supported by sound evidence. Since the development of these evidence-based medicine lists following a scientific procedure which includes evidence searching and implementation from academic literature, these medicines supported by the evidence-based medicine lists will be not referred to published research paper for further assessment (No need to next step). However, if certain medicine included in NRDL of UEBMI was only included in one or none of these three lists, for considering many guidelines do not purport to provide an exhaustive list of all effective medicines, it will be referred to some published research paper with high-level evidence grade for further assessment.

Step two, collect evidence from systematic review or meta-analysis. For the medicines only included in one or none of these three international evidence-based lists, the systematic review or meta-analysis on their antihypertensive effectiveness will be reviewed. If certain medicine was confirmed effective in treating hypertension with low incidence of adverse events in meta-analysis or systematic review with acceptable literature quality, the effectiveness of this medicine can be also considered as having sound evidence support. Since systematic reviews or meta-analyses are the literature with highest evidence grade for its comprehensively combining the results of previous RCT, these medicines supported by meta-analysis or systematic review will be not referred to published RCT for further assessment (No need to next step). However, for the rest medicines having evidence support from neither international evidence-based medicine lists nor meta-analyses / systematic reviews, they will be referred to published RCT for further assessment.

Step three, collect evidence from RCT. For the rest medicines mentioned above, RCTs on their effectiveness in treating hypertension will be reviewed. If certain medicine was reported by RCT with acceptable methodological quality as an effective antihypertensive medication with few adverse events, the effectiveness of this medicine can also be considered as having sound evidence support. Or else, the effectiveness of certain medicine in treating hypertension will be considered as having insufficient evidence.

### Statistical analysis

The times of certain medicine included in the three-international evidence-based clinical guidelines on hypertension were calculated. For the medicines only included in one guideline or not included in any one of these three guidelines, the numbers of the kinds of medicines supported by published systematic review, meta-analysis and RCT were calculated respectively.

## Results

### General information

There are 97 antihypertensive medicines included in NRDL of UEBMI, which were divided into Class A and Class B. There are 26 medicines in Class A (26.8%) and 71 included in Class B (73.2%). Based on the mechanism of action, the 97 antihypertensive medicines can be divided into seven categories, such as calcium antagonist, β-blockers, diuretics, angiotensin converting enzyme inhibitors (ACEIs), Angiotensin II receptor antagonist, vasodilators and others, respectively accounting for 24.7, 14.4, 11.4, 15.5, 12.4, 7.2 and 14.4% of all the 97 kinds of medicines.

### Evidence from the three international evidence-based clinical guidelines

Twenty-six of the 97 surveyed medicines were included in all or two of the three international evidence-based clinical guidelines, which accounted for 26.8%. The numbers of the kinds of medicines included in one of the three clinical guidelines were 16, accounting for 16.5% of the total. Additionally, fifty-five kinds of medicines (approximately 60%) were included in neither of the guidelines (Shown in Table 1).

**Table 1:** Evidence for the effectiveness of anti-hypertensive drugs in NRDL of UEBMI——From the international evidence-based drug list

	Dosage form	JNC 8	NICE 2011	WHO EML 2017	Frequency of inclusion
Calcium antagonist					
Class A					
Amlodipine	oral release dosage form	√	√	√	3
Nimodipine	oral release dosage form	–	–	–	0
Nitrendipine	oral release dosage form	–	–	–	0
Nifedipine	oral release dosage form	–	√	√	2
Diltiazem	oral release dosage form	–	√	√	2
Verapamil	oral release dosage form	–	√	√	2
Verapamil	injection	–	–	√	1
Class B					
L - nmda amlodipine	oral release dosage form	–	–	–	0
Amlodipine atorvastatin calcium	oral release dosage form	–	–	–	0
Benidipine	oral release dosage form	–	–	–	0
Felodipine	oral release dosage form, controlled release dosage form	–	√	–	1
Felodipine II	controlled release dosage form	–	√	–	1
Lacidipine	oral release dosage form	–	√	–	1
Lercanidipine	oral release dosage form	–	√	–	1
Nicardipine	oral release dosage form, controlled release dosage form	–	–	–	0
Nicardipine	injection	–	–	–	0
Nimodipine	injection	–	–	–	0
Nitrendipine and Atenolol	oral release dosage form	–	–	–	0
Cilnidipine	oral release dosage form	–	–	–	0
Nifedipine (I,II,III)	controlled release dosage form	–	√	√	2
Levamlodipine besylate tablets	oral release dosage form	–	√	–	1
Diltiazem	injection	–	–	–	0
Diltiazem (II)	controlled release dosage form	–	√	√	2
Verapamil	controlled release dosage form	–	√	√	2
β blockers					
Class A					
Propranolol	oral release dosage form	–	√	√	2
Atenolol	oral release dosage form	√	√	–	2
Bisoprolol	oral release dosage form	–	√	√	2
Metoprolol	oral release dosage form	√	√	–	2
Metoprolol	injection	–	–	–	0
Class B					
Propranolol	Sustained or controlled release dosage form	–	√	√	2
Propranolol	injection	–	–	–	0
Sotalol	oral release dosage form	–	√	–	1
Sotalol	injection	–	–	–	0
Esmolol	injection	–	–	–	0
Metoprolol	controlled release dosage form	√	√	–	2
Arotinolol	oral release dosage form	–	–	–	0
Carvedilol	oral release dosage form	–	–	–	0
Labetalol	oral release dosage form	–	–	–	0
diuretics					
Class A					
Hydrochlorthiazide	oral release dosage form	√	√	√	3
Indapamide	oral release dosage form, controlled release dosage form	√	√	–	2
Furosemide	oral release dosage form	–	–	√	1
Furosemide	injection	–	–	√	1
Triamterene	oral release dosage form	–	–	–	0
Spironolactone	oral release dosage form	–	√	√	2
Class B					
Bumetanide	oral release dosage form	–	–	–	0
Bumetanide	injection	–	–	–	0
Torasemide	oral release dosage form	–	–	–	0
Torasemide	injection	–	–	–	0
Amiloride	oral release dosage form	–	√	√	2
ACEIs					
Class A					
Captopril	oral release dosage form	√	√	–	2
Enalapril	oral release dosage form	√	√	√	3
Class B					
Benazepril	oral release dosage form	–	–	–	0
Fosinopril	oral release dosage form	–	–	–	0
Lisinopril	oral release dosage form	√	√	–	2
Ramipril	oral release dosage form	–	√	–	1
Imidapril	oral release dosage form	–	–	–	0
Perindopril	oral release dosage form	–	√	–	1
Cilazapril	oral release dosage form	–	–	–	0
Amlodipine benapril I (II)	oral release dosage form	–	–	–	0
Benapril hydrochlorothiazide	oral release dosage form	–	–	–	0
compound captopril	oral release dosage form	–	–	–	0
Lynopli hydrochlorothiazide	oral release dosage form	–	–	–	0
Enalpril hydrochlorothiazide	oral release dosage form	–	–	–	0
Enopril folic acid	oral release dosage form	–	–	–	0
Angiotensin II receptor antagonist					
Class B					
Olmesartan Medoxomil	oral release dosage form	–	–	–	0
Irbesartan	oral release dosage form, controlled release dosage form	√	√	–	2
Candesartan	oral release dosage form	√	√	–	2
Losartan	oral release dosage form	√	√	√	3
Telmisartan	oral release dosage form	√	√	–	2
Valsartan	oral release dosage form	√	√	–	2
Omethane hydrochlorothiazide	oral release dosage form	–	–	–	0
irbesartan hydrochlorothiazide	oral release dosage form	–	–	–	0
chlorothiazide	oral release dosage form	–	–	–	0
Timisaltan hydrochlorothiazide	oral release dosage form	–	–	–	0
Valsartan chlordipine I (II)	oral release dosage form	–	–	–	0
Valsartan hydrochlorothiazide	oral release dosage form	–	–	–	0
vasodilators					
Class A					
Phentolamine	injection	–	–	–	0
Sodium nitroprusside	injection	–	–	√	1
Class B					
Phenoxybenzamine	oral release dosage form	–	–	–	0
Phenoxybenzamine	injection	–	–	–	0
Hydralazine	oral release dosage form	–	–	√	1
Hydralazine	injection	–	–	√	1
Minoxidil	oral release dosage form	–	–	–	0
Others					
Class A					
Reserpine	injection	–	–	–	0
Prazosin	oral release dosage form	–	√	–	1
Compound reserpine	oral release dosage form	–	–	–	0
Compound reserpine ammonia benzene pteridine	oral release dosage form	–	–	–	0
Class B					
Dibazole	oral release dosage form	–	–	–	0
Methyldopa	oral release dosage form	–	√	√	2
Clonidine	oral release dosage form	–	–	–	0
Clonidine	Patch	–	–	–	0
Reserpine	oral release dosage form	–	–	–	0
Ligustrazine	Injection	–	–	–	0
Doxazosin	oral release dosage form, controlled release dosage form	–	√	–	1
Naftopidil	oral release dosage form	–	–	–	0
Urapidil	oral release dosage form, controlled release dosage form	–	–	–	0
Urapidil	injection	–	–	–	0

### Evidence from published literature with high evidence grade

For the 71 kinds of medicines included in one or none of the three international evidence-based clinical guidelines, twenty-six of them had been evaluated in systematic review or meta-analysis, while 12 of them assessed in RCT. However, the other 33 medicines had not been reported in published literature with high evidence grade as mentioned above.

### Evidence from systematic review and meta-analysis

For all the 25 systematic reviews and meta-analyses initially included, their PRISMA scores were more than 21 points, which infers that the literature integrity can be considered fairly acceptable (See column 5, Table 2). With respect to the methodological quality assessed by the AMSTAR scale, the AMSTAR scores were between 7 and 11 points for all the 25 included systematic reviews and meta-analyses, which can be considered that their methodological quality were good (Shown in column 4, Table 2). And the completion of each items in AMSTAR Scale are shown in Additional file 1: Table S1.

**Table 2:** Evidence for effectiveness of anti-hypertensive medicines in NRDL of UEBMI——From systematic review and meta-analysis

	Dosage form	Meta-analysis /Systematic review (First author, year)	AMSTAR score	PRISMA score	Research conclusions	Sound support for effectiveness (Y / N)
Calcium antagonist						
Class A						
Nimodipine	oral release dosage form	Chen Q, 2014 [27]	8.5	25	For patients with HICH, nimodipine combined with edaravone has significant clinical efficacy in the treatment of hypertensive cerebral hemorrhage, and can improve the functional rehabilitation of the nerves and effectively reduce the volumes of intracranial hematoma and peripheral edema.	Y
Nitrendipine	oral release dosage form	Du X, 2014 [32]	8	21	In lowering blood pressure, Amlodipine is better than Nitrendipine in the cost and effect.	N
Class B						
Levamlodipine besylate	oral release dosage form	Zhao Z, 2015 [26]	8.5	24	Levamlodipine is more effective and safer than amlodipine in treatment of mild to moderate hypertension than, which is thus worthy of clinical application.	Y
Felodipine	oral release dosage form, controlled release dosage form	Zhang T, 2013 [33]	8	21	In the treatment of Chinese patients with hypertension, amlodipine both in reducing systolic or diastolic pressure were better than felodipine, no statistical significance on both heart rate and adverse drug reaction differences.	N
Felodipine II	controlled release dosage form	Zhang T, 2013 [33]	8	21	In the treatment of Chinese patients with hypertension, amlodipine both in reducing systolic or diastolic pressure were better than felodipine, no statistical significance on both heart rate and adverse drug reaction differences.	N
Lercanidipine	oral release dosage form	Ran Y, 2015 [20]	8.5	23	Based on the current evidence, the safety and compliance of lercanidipine is better than amlodipine in the treatment of mild to moderate hypertension.	Y
Nicardipine	injection	Jiang C, 2013 [19]	8.5	24.5	Nicardipine was safe and effective in the treatment of hypertensive emergency.	Y
Lacidipine	oral release dosage form	Hua Q, 2014 [18]	9.5	23	Lacidipine has been similar to amlodipine in the treatment of essential hypertension with less adverse drug reaction.	Y
Cilnidipine	oral release dosage form	Li S, 2012 [16]	9	22.5	Cilnidipine has the similar efficacy and safety compared with control group in treating essential hypertension.	Y
β-blockers						
Class B						
Arotinolol	oral release dosage form	Du B, 2009 [21]	8.5	21.5	In treating essential hypertension, there is no significant difference in efficacy and safety between Arotinolol and control group, such as Cilnidipine, felodipine and Imidapril.	Y
Labetalol	oral release dosage form	Magee LA, 2015 [29]	9	24	Labetalol is a reasonable choice for treatment of severe or non-severe hypertension in pregnancy	Y
Carvedilol	oral release dosage form	Chen S, 2015 [13]	9	24	Carvedilol has a greater portal hypertensive effect than propranolol. Further comparative trials of the two drugs are required to identify the effect of MAP reduction.	Y
Esmolol	injection	Garnockjones KP, 2012 [31]	10	25	Definitive conclusions on the efficacy of esmolol are difficult to reach, as most trials investigating esmolol have limitations such as small patient populations, and few studies investigate the same parameters.	N
ACEIs						
Class B						
Perindopril	oral release dosage form	Gasowski J, 2010 [12]	8.5	24	Perindopril is an effective antihypertensive medication. Seems not to be adversely affected by the clinical profile of the patient.	Y
Benazepril	oral release dosage form	Zhao S, 2015 [22]	7.5	22	Benazepril can more effectively lower the blood pressure of patients with primary hypertension than captopril.	Y
Fosinopril	oral release dosage form	Zeng X, 2014 [11]	8.5	23.5	The curative effect of fosinopril is almost the same as calcium antagonists in the treatment of mild to moderate essential hypertension, but superior to other types of antihypertensive drugs and shows good safety.	Y
Enopril folic acid	oral release dosage form	Zhang Y, 2015 [23]	7.5	22	Enopril folic acid showed better efficacy in lowering blood pressure and preventing cardiovascular accident than Enopril.	Y
Vasodilators						
Class A						
Sodium nitroprusside	injection	Dong W, 2012 [17]	9.5	22	Sodium nitroprusside is effective for the treatment of hypertensive emergency, while the ADRs are acceptable.	Y
Class B						
Hydralazine	oral release dosage form	Kandler M R, 2010 [34]	11	27	Hydralazine may reduce blood pressure when compared to placebo in patients with primary hypertension, however this data is based on before and after studies, not RCTs. Furthermore, its effect on clinical outcomes remains uncertain.	N
Angiotensin II receptor antagonist						
Class B						
Irbesartan /Hydrochlorothiazide	oral release dosage form	Wu H, 2011 [24]	8.5	23	Irbesartan/hydrochlorothiazide combination therapy is more effective than control group in treating essential hypertension and there is no significant difference in safety.	Y
Valsartan /Hydrochlorothiazide	oral release dosage form	Jin J, 2013 [25]	8	21	In the treatment of primary hypertension, Valsartan /Hydrochlorothiazide combination has better anti-hypertension efficacy with less adverse drug reaction.	Y
Others						
Class A						
Compound reserpine	oral release dosage form	Hu L, 2012 [30]	9	25.5	Based on current research evidence, Compound reserpine tablets are safe and effective.	Y
Compound reserpine ammonia benzene pteridine	oral release dosage form	Wu Y, 2009 [28]	9	23	Compound reserpine ammonia benzene pteridine appears to have the same effect as other anti-hypertensive drugs without having more adverse events.	Y
Class B						
Doxazosin	oral release dosage form	Ke Z, 2015 [14]	8.5	24	Doxazosin has affirmed effect on mild to moderate essential hypertension, with little side effect, good patient tolerance, especially for hypertension patients with benign prostatic hyperplasia.	Y
Urapidil	injection	Zhou X, 2016 [15]	7.5	21.5	Urapidil and conventional vasedilator like Nitroglycerin are effective to heart failure with hypertension. For lowering blood pressure, their efficacy are equal, but for reducing patients BNP and heart rate, Urapidil is better than the other.	Y
Reserpine	oral release dosage form	Shamon S D, 2009 [35]	9	25	Reserpine is effective in reducing SBP roughly to the same degree as other first-line antihypertensive drugs. However, we could not make definite conclusions regarding the dose-response pattern because of the small number of included trials. More RCTs are needed to assess the effects of reserpine on blood pressure and to determine the dose-related safety profile before the role of this drug in the treatment of primary hypertension can be established.	N

The results published in these systematic review and meta-analysis indicated that fosinpril [15], perindopril [16], carvedilol [17], Doxazoxin [18], Urapidil (injection) [19], Cilnidipine [20], Sodium nitroprusside [21], Lacidipine [22], Nicardipine (injection) [23], Lercanidipine [24], Arotinolol [25], Benazepril [26], Enopril folic acid [27], Irbesartan hydrochlorothiazide [28], Valsartan hydrochlorothiazide [29], Levamlodipine besylate [30], Nimodipine (oral dosage) [31], Compound reserpine ammonia benzene pteridine [32], Labetalol [33], and Compound reserpine [34] were fairly effective in treating hypertension with low incidence rate of adverse reactions. In other words, the effectiveness of regarding medicines were supported by these systematic review and meta-analysis. However, for Esmolol [35], Nitrendipine [36], Felodipine [37], Felodipine II [37], Hydralazine [38] and Reserpine [39], their effectiveness in treating hypertension were not confirmed in regarding systematic review or meta-analysis. For instance, the reported results pointed out that the data on efficacy and safety of Hydralazine was based on before and after studies, not RCTs. The efficacy of Hydralazine in treating hypertension remains uncertain [38]. (See Table 2).

### Evidence from RCT

Fourteen RCTs were initially included in this study and their methodological qualities were assessed by the adjusted Jadad scale. Eleven of them had the Jadad score not less than 3 points, while the other three had the scores between 0 and 2 points. (See Additional file 2: Table S2).

Although the safety and effectiveness of Amlodipine benapril [40], Metoprolol (injection) [41], were reported in some RCTs, the Jadad score of these RCTs were all between 0 and 2 points, which were considered as unacceptable methodological quality. Thus, the conclusions of these studies should be cautious to use, which implies uncertainty of the effectiveness of these medicines. For Propranolol (injection), its effectiveness of treating hypertension was also confirmed by one RCT published in 2007 [42], though another one on its effectiveness published in 2001 has unacceptable methodological quality [43]. Additionally, as reported by the other 10 RCTs with Jadad score not less than 3 points. Furosemide (injection) [44], Torasemide (injection) [45], Naftopidil [46], Olmesartan Medoxomil [47], Ramipril [48, 49], Imidapril [50], Diltiazem (injection) [51], Enalpril hydrochlorothiazide [52] and Cilazapril [53] were fairly effective in treating hypertension with few adverse events. Therefore, it can be considered that the effectiveness of these five medicines were supported by RCTs with acceptable methodological quality. (See Table 3).

**Table 3:** Evidence for effectiveness of anti-hypertensive medicines in NRDL of UEBMI——From RCT

	Dosage form	RCT (First author, year)	JADAD score	Research conclusions	Sound support for effectiveness (Y / N)
ACEIs					
Class B					
Amlodipine benapril	oral release dosage form	Cao J, 2012 [36]	2	Amlodipine benapril can achieve better antihypertensive effect and reduce adverse reactions, which is worthy of clinical promotion.	N
Enalpril hydrochlorothiazide	oral release dosage form	Wang X, 2006 [48]	4	The efficacy of combination of enalapril hydrochlorothiazide is better than single use of enalapril in the treatment of mild and moderate primary hypertension. Combination of enalapril hydrochlorothiazide has the same safety and toleration as enalapril.	Y
Imidapril	oral release dosage form	Jiang X, 2005 [46]	4	Imidapril and Benapril are both similarly effective in the reduction of the peripheral blood pressure and the central blood pressure.	Y
Ramipril	oral release dosage form	Tao B, 2006 [44]	4	The combination of irbesartan with felodipine or ramipril showed synergist antihypertensive effects. Moreover, the combination of irbesartan with felodipine was superior to combination of irbesartan with ramipril.	Y
		Rokoss M J, 2005 [45]	5	Beneficial effects of ramipril are observed in the treatment of hypertension and congestive heart failure, prevention of cardiovascular events in high-risk patients, prevention of congestive heart failure, diabetes and other vascular events	Y
Cilazapril	oral release dosage form	Schiffrin EL, 2008 [49]	4	These results may indicate that treatment with cilazapril and perhaps with other angiotensin-I- converting enzyme inhibitors as well may improve the clinical outcome in hypertension by inducing a regression of abnormal resistance vessel structure and function.	Y
β-blockers					
Class A					
Metoprolol	injection	Lu N, 2006 [37]	2	Metoprolol is effective and safe for patients with unstable angina pectoris. It may significantly lower the risk of refractory angina pectoris.	N
Class B					
Propranolol	injection	Jiang X, 2001 [39]	2	Combination of propranolol with prazosin caused a signincantly greater reduction in the portal pressure on the third month and the reduction in H/L and responding rate were greater in the treatment group than in the control group in patients with a previous bleeding episode.	N
		Zuo W, 2007 [38]	3	Propranolol, ISMN and spironolactone in combination can effectively prevent the occurrence of hemorrhage in cirrhotic patients with esophageal varices	Y
Calcium antagonist					
Class B					
Diltiazem	injection	Collaborative Group of Diltiazem, 2005 [47]	4	Intravenous diltiazem therapy is effective and safe for patients with unstable angina pectoris. It may significantly lower the risk of refractory angina pectoris compared with intravenous nitroglycerin.	Y
Diuretics					
Class A					
Furosemide	injection	Huang G, 2008 [40]	3	The use of 125 ml 20% mannitol each time plus 20 mg furosemide is more reasonable than other combinations. Meanwhile, semis mannitol combined with moderate or large dose of albumin has certain advantages too.	Y
Class B					
Torasemide	injection	Zheng W, 2008 [41]	3	Torasemide injection is an effective and safe drug for the treatment of congestive heart failure with edema.	Y
Angiotensin II receptor antagonist					
Class B					
Olmesartan Medoxomil	oral release dosage form	Liao Y, 2014 [43]	3	Compared with enalapril, Olmesartan Medoxomil has more significant inhibition and reversal effects of left ventricular remodeling in treatment of the morning surge of hypertension.	Y
Others					
Class B					
Naftopidil	oral release dosage form	Lu Q, 2000 [42]	3	The efficacy of Naftopidil in controlling the blood pressure is as efficient as that of Terazosin and both drugs are well tolerated by the patients.	Y

### Overall situation of evidence support

Taking the evidence from three international evidence-based clinical guidelines on hypertension, systematic review/meta-analysis, and RCT, as well as the methodological quality of the literature comprehensively into account, the effectiveness of 56 kinds of medicines were supported by fairly sound evidence. To be specific, twenty-six of the medicines were supported by international evidence-based clinical guideline on hypertension, while twenty were supported by systematic review/meta-analysis and the other ten supported by RCT. However, for the other 41 kinds of medicines, two out of them were supported by RCTs with poor methodological quality, thirty-three of them did not gain support from the evidence with high grade, and six medicines were reported uncertainty of antihypertensive effectiveness in meta-analysis or systematic review. (See Table 4).

**Table 4:** Overall situation of evidence support for the effectiveness of anti-hypertensive medicines in NRDL

	Sound evidence support			Subtotal	Insufficient evidence support			Subtotal
	Support from international evidence-based drug list	Support from systematic review or meta-analysis	Support from RCT		Reported by RCT with poor quality	No evidence involved	No definite conclusion in systematic review or meta-analysis	
Calcium antagonist	7 (29.2%)	6 (25.0%)	1 (4.2%)	14 (58.3%)	0 (0.0%)	7 (29.2%)	3(12.5%)	10 (41.7%)
β blockers	6 (42.9%)	3 (21.4%)	1 (7.1%)	10 (71.4%)	1 (7.1%)	2 (14.3%)	1 (7.1%)	4 (28.6%)
Diuretics	4 (36.4%)	0 (0.0%)	2(18.2%)	6 (54.6%)	0 (0.0%)	5 (45.4%)	0 (0.0%)	5 (45.4%)
ACEIs	3 (20.0%)	4 (26.7%)	4(26.7%)	11 (73.3%)	1 (6.7%)	3 (20.0%)	0 (0.0%)	4 (26.7%)
Angiotensin II receptor antagonist	5 (41.7%)	2 (16.6%)	1 (8.3%)	8 (66.7%)	0 (0.0%)	4 (33.3%)	0 (0.0%)	4 (33.3%)
Vasodilators	0 (0.0%)	1 (14.3%)	0 (0.0%)	1 (14.3%)	0 (0.0%)	5 (71.4%)	1 (14.3%)	6 (85.7%)
Others	1 (7.1%)	4 (28.6%)	1 (7.1%)	6 (42.9%)	0 (0.0%)	7 (50.0%)	1 (7.1%)	8 (57.1%)
Class A	13 (50.0%)	4 (15.4%)	1 (3.8%)	18 (69.2%)	1 (3.8%)	6 (23.1%)	1 (3.8%)	8 (30.8%)
Class B	13 (18.3%)	16 (22.6%)	9 (12.7%)	38 (53.5%)	1 (1.4%)	27 (38.1%)	5 (7.0%)	33 (46.5%)
Total	26 (26.8%)	20 (20.6%)	10 (10.3%)	56 (57.7%)	2 (2.1%)	33 (34.0%)	6 (6.2%)	41 (42.3%)

With regard to the antihypertensive medicines with different major functions, the evidence support status was also very different among them. The proportion of the medicines with confirmed effectiveness ranged from 14.3% (for Vasodilators) to 73.3% (for ACEIs). Regarding to the different management categories, approximately 70% of the antihypertensive medicines in Class A have sound evidence support for their effectiveness, while the corresponding proportion is about 50% for the antihypertensive medicines in Class B. (See Table 4).

## Discussion

This study applied a rapid evaluation strategy to determine the situation of evidence support for the effectiveness of medicines. It reveals that there is insufficient evidence of effectiveness for approximately 45% of the antihypertensive medicines included in the NRDL of UEBMI. The results with policy implication will provide a reference for the adjustment and improvement of the NRDL. Moreover, a series of factors leading to these results deserved further consideration.

Firstly, since the JNC8, NICE 2011, and EML 2017 are all evidence-based drug lists, the great differences between NRDL of UEBMI and three international evidence-based drug lists can not be ignored. To explore the underlying reasons for such discordance, the regional diversity may be the plausible. For population differences in response to antihypertensive treatments, the decision-makers may include other medicines in the NRDL to meet local needs, and subsequently lead to large differences between NRDL and international evidence-based drug list. However, whether the differences are reasonable still remains to be intensively determined.

To this point, it is important to fully examine published literature with high evidence grade, such as meta-analysis, systematic review, and RCT. In this study, 20.6% (20/97) of antihypertensive medicines were supported by meta-analysis or systematic review with regard to their effectiveness, while only 10.3% (10/97) of antihypertensive medicines were confirmed by RCT. Besides, it is worth noting that nearly 30% of the included RCTs had unacceptable quality, which implied that the methodological quality of RCT needs further improvement. Some shortcomings, such as inappropriate method in description of withdrawals and dropouts and failure in double blinding, had greatly deteriorated the authenticity and reliability of RCT. For the sake of further improving the methodological or report quality of systematic review, meta-analysis and RCT, it is recommended to provide researchers with methodological trainings on special issues, such as proper quality evaluation methods for meta-analysis, systematic review and RCT, appropriate method for applying randomization and blinding [54]. Additionally, for ensuring high quality of scientific evidence, strict monitoring on methodology as well as report quality of submitted manuscript before its publication is strongly recommended.

With regard to the overall situation of evidence support for the effectiveness, only a small proportion of the medicines among vasodilators were confirmed by sound evidence, which highlights the need of further adjustment for certain kinds of medicines. Additionally, in comparison with Class B, a larger proportion of antihypertensive medicines in Class A were confirmed by sound evidence, which demonstrates that more deliberate management had been implemented on essential and commonly used medicines.

However, it is still noteworthy that there is insufficient evidence for the effectiveness of almost 45% of the antihypertensive medicines in the NRDL of UEBMI. What is more, six of them were demonstrated by meta-analysis or systematic review as having no definitive conclusion on the effectiveness of treating hypertension. Although it can be more prudent to wait for more evidence, these medicines were still included in the NRDL. There are some reasons underlying this fact. One plausible reason may be the price issue even though it is not the focus of this paper. There is no denying that the drugs with less efficacies but lower prices also tended to be included in the insurance drug list for their potential great economic accessibility. Besides, the influence of the decision-making process of medicine inclusion in China also can not be ignored. It is an expert reviews system with hundreds or even thousands of experts involved to provide their opinions on the safety, clinical efficacy and economic cost of certain medicine. And the decision will be made to include or exclude certain medicine from the NRDL by comprehensively taking the experts’ opinion into account [55]. Apparently, this decision-making process mainly depends on the expert opinion and seems more efficient than the process of “evidence collection—evidence evaluation—evidence application—decision-making”. However, since it can hardly identify whether the expert opinions were based on “evidence” rather than “experience”, its potential risk of decision-making error would be much higher. For the timeliness of decision-making and the practicality of medical practice, it will be more sensible to combine expert opinion with scientific evidence [56].

To promote scientific and efficient decision-making, some recommendations are also proposed as two folds: on the one hand, we should steadily improve the methodological quality of academic literature, such as meta-analysis, systematic review and RCT. It is also essential to widely implement continuing education and training for evaluation methodology of medicine safety, efficacy and economy. On the other hand, we should set up high quality evidence database and establish specialized evidence evaluation agencies [57]. These databases should be open to medical institutions, decision-making departments, and even the public for timely access to scientific evidence of medicines and other health technologies [58]. On the basis of availability of high quality evidences, concerned agencies will be likely to standardize the decision-making procedures for the inclusion of medicines into the NRDL, and formulate evidence evaluation methods and technical guidelines [59]. All these will facilitate the decision-making supervision and promote scientific and evidence-based decision-making.

Although this research and the findings are China-based, the policy implications probably have wider applicability in the sense that many other countries are also confronted with similar dilemma of decision-making while including medicines into NRDL. Even more important, this study provides a new strategy of comprehensively integrating international and local available high-grade evidence to make a rapid evaluation of NRDL’s evidence-based rationality. Apparently, this strategy is not unique to a particular country or only applicable to single kinds of medicine. It can also be widely applied by many other countries to examine the evidence for certain kinds of medicines, such as antihypertensive medicines, anti-diabetic medicines, anti-cancer medicines and so on.

As a preliminary application of a rapid evaluation strategy, this study still has some limitations, which should be noticed when subsequently quoting the results or applying the evaluation strategy. Firstly, this study mainly collected published meta-analysis, systematic review, and RCT to evaluate the effectiveness of the medicines without direct evidence support from international evidence-based drug lists. However, the publication bias is still hard to be completely ruled out. Secondly, by considering scientific evidence support and representativeness, three international evidence-based medicine lists were selected as reference criteria in this study. However, there is still doubt about whether there are other international evidence-based medicine lists more appropriately for the reference criteria. In view of this issue, the intensive comparison between many commonly used international evidence-based medicine lists will be further considered in future studies. And the research outcomes will further improve the rapid evaluation strategy in this study.

## Conclusions

Nearly 45% of the antihypertensive medicines included in the NRDL of UEBMI lack sound evidence supports for its effectiveness. Some shortcomings in reporting and methodological quality of published meta-analysis, systematic review, and RCT had deteriorated their authenticity and reliability, thus weakening their evidence support for certain antihypertensive medicines. It highlights the importance of providing professional and comprehensive methodological trainings to researchers and reinforcing methodological quality assurance in writing peer-reviewed journal articles. To optimize the selection of medicines into NRDL, it is recommended to establish specialized evidence evaluation agencies and set up high quality evidence database to timely provide sound evidence, as well as standardize the decision-making procedures for inclusion of medicines.

## Additional files

Additional file 1: **Table S1.** The completion of each items in AMSTAR Scale——For the included systematic reviews and meta-analyses. It provides information on whether certain included systematic reviews and meta-analyses had completed each of the 11 items in AMSTAR Scale. (DOCX 27 kb)

Additional file 2: **Table S2.** Jadad score for the included randomized controlled trials. It provides information on whether certain included RCT had completed each of the 3 items, namely “randomization”, “double blinding” and “withdrawals and dropouts”, in the Jadad scale. (DOCX 21 kb)

## Abbreviations

ACEIs: Angiotensin Converting Enzyme Inhibitors
JNC 8: the Eighth Report of the Joint National Committee on Prevention, Detection, Evaluation and Treatment of high blood pressure
NICE 2011: the Clinical Management of Primary Hypertension in Adults, 2011 version
NRDL: National Reimbursement Drug List
UEBMI: Urban Employee Basic Medical Insurance
WHO EML 2017: World Health Organization Essential Medicine List of 2017 version
