# Supplementary material for: Evidence for the effectiveness of anti-hypertensive medicines included on the Chinese National Reimbursement Drug List
Source: BMC Health Serv Res. 2019 Feb 11;19:112. doi: 10.1186/s12913-019-3937-0 (PMC6369556; doi:10.1186/s12913-019-3937-0)
Supplement: Supplementary file 1 — Table S1. The completion of each items in AMSTAR Scale——For the included systematic reviews and meta-analyses. It provides information on whether certain included systematic reviews and meta-analyses had completed each of the 11 items in AMSTAR Scale. (DOCX 27 kb) [file 12913_2019_3937_MOESM1_ESM.docx]

Additional file 1: **Table S1** The completion of each items in AMSTAR Scale——For the included systematic reviews and meta-analyses

|  | Dosage form | First author, year | AMSTAR score | Items | | | | | | | | | | |
| --- | --- | --- | --- | --- | --- | --- | --- | --- | --- | --- | --- | --- | --- | --- |
|  |  |  |  | 1^a^ | 2^b^ | 3^c^ | 4^d^ | 5^e^ | 6^f^ | 7^g^ | 8^h^ | 9^i^ | 10^j^ | 11^k^ |
| **Calcium antagonist** |  |  |  |  |  |  |  |  |  |  |  |  |  |  |
| **Class A** |  |  |  |  |  |  |  |  |  |  |  |  |  |  |
| Nimodipine | oral release dosage form | Chen Q, 2014 [27] | 8.5 | N | Y | Y | Y | P | Y | Y | Y | Y | Y | N |
| Nitrendipine | oral release dosage form | Du X, 2014 [32] | 8 | N | Y | Y | Y | P | P | Y | Y | Y | Y | N |
| **Class B** |  |  |  |  |  |  |  |  |  |  |  |  |  |  |
| Levamlodipine besylate | oral release dosage form | Zhao Z, 2015 [26] | 8.5 | N | Y | Y | Y | P | Y | Y | Y | Y | Y | N |
| Felodipine | oral release dosage form, controlled release dosage form | Zhang T, 2013 [33] | 8 | N | Y | Y | P | P | Y | Y | Y | Y | Y | N |
| Felodipine Ⅱ | controlled release dosage form | Zhang T, 2013 [33] | 8 | N | Y | Y | P | P | Y | Y | Y | Y | Y | N |
| Lercanidipine | oral release dosage form | Ran Y, 2015 [20] | 8.5 | N | Y | Y | Y | P | Y | Y | Y | Y | Y | N |
| Nicardipine | injection | Jiang C, 2013 [19] | 8.5 | N | Y | Y | Y | P | Y | Y | Y | Y | Y | N |
| Lacidipine | oral release dosage form | Hua Q, 2014 [18] | 9.5 | N | Y | Y | Y | P | Y | Y | Y | Y | Y | Y |
| Cilnidipine | oral release dosage form | Li S, 2012 [16] | 9 | N | Y | Y | Y | P | Y | P | Y | Y | Y | Y |
| **β-blockers** |  |  |  |  |  |  |  |  |  |  |  |  |  |  |
| **Class B** |  |  |  |  |  |  |  |  |  |  |  |  |  |  |
| Arotinolol | oral release dosage form | Du B, 2009 [21] | 8.5 | N | Y | Y | P | P | Y | Y | Y | Y | P | Y |
| Labetalol | oral release dosage form | Magee LA, 2015 [29] | 9 | N | Y | Y | Y | P | Y | Y | Y | Y | Y | N |
| Carvedilol | oral release dosage form | Chen S, 2015 [13] | 9 | N | Y | Y | Y | P | Y | P | Y | Y | Y | Y |
| Esmolol | injection | Garnockjones KP, 2012 [31] | 10 | N | Y | Y | Y | Y | Y | Y | Y | Y | Y | Y |
| **ACEIs** |  |  |  |  |  |  |  |  |  |  |  |  |  |  |
| **Class B** |  |  |  |  |  |  |  |  |  |  |  |  |  |  |
| Perindopril | oral release dosage form | Gasowski J, 2010 [12] | 8.5 | N | N | Y | Y | P | Y | Y | Y | Y | Y | Y |
| Benazepril | oral release dosage form | Zhao S, 2015 [22] | 7.5 | N | Y | Y | Y | N | Y | P | Y | Y | Y | N |
| Fosinopril | oral release dosage form | Zeng X, 2014 [11] | 8.5 | N | Y | Y | Y | P | Y | Y | Y | Y | Y | N |
| Enopril folic acid | oral release dosage form | Zhang Y, 2015 [23] | 7.5 | N | Y | Y | Y | N | Y | P | Y | Y | Y | N |
| **Vasodilators** |  |  |  |  |  |  |  |  |  |  |  |  |  |  |
| **Class A** |  |  |  |  |  |  |  |  |  |  |  |  |  |  |
| Sodium nitroprusside | injection | Dong W, 2012 [17] | 9.5 | N | Y | Y | Y | P | Y | Y | Y | Y | Y | Y |
| **Class B** |  |  |  |  |  |  |  |  |  |  |  |  |  |  |
| Hydralazine | oral release dosage form | Kandler M R, 2010 [34] | 11 | Y | Y | Y | Y | Y | Y | Y | Y | Y | Y | Y |
| **Angiotensin Ⅱ receptor antagonist** |  |  |  |  |  |  |  |  |  |  |  |  |  |  |
| **Class B** |  |  |  |  |  |  |  |  |  |  |  |  |  |  |
| Irbesartan /Hydrochlorothiazide | oral release dosage form | Wu H, 2011 [24] | 8.5 | N | Y | Y | Y | P | Y | Y | Y | Y | Y | N |
| Valsartan /Hydrochlorothiazide | oral release dosage form | Jin J, 2013 [25] | 8 | N | Y | Y | Y | P | P | Y | Y | Y | Y | N |
| **Others** |  |  |  |  |  |  |  |  |  |  |  |  |  |  |
| **Class A** |  |  |  |  |  |  |  |  |  |  |  |  |  |  |
| Compound reserpine | oral release dosage form | Hu L, 2012 [30] | 9 | N | Y | Y | Y | P | P | Y | Y | Y | Y | Y |
| Compound reserpine ammonia benzene pteridine | oral release dosage form | Wu Y, 2009 [28] | 9 | N | Y | Y | Y | P | Y | P | Y | Y | Y | Y |
| **Class B** |  |  |  |  |  |  |  |  |  |  |  |  |  |  |
| Doxazosin | oral release dosage form | Ke Z, 2015 [14] | 8.5 | N | Y | Y | Y | P | Y | Y | Y | Y | Y | N |
| Urapidil | injection | Zhou X, 2016 [15] | 7.5 | N | Y | Y | P | P | Y | P | Y | Y | Y | N |
| Reserpine | oral release dosage form | Shamon S D, 2009 [35] | 9 | N | Y | Y | Y | Y | Y | Y | Y | Y | Y | N |

^*^ Y, “Yes”; P, “Partly”; N, “No”.

^a^ Item 1, “Was an ‘a priori’ design provided?”

^b^ Item 2, “Was there duplicate study selection and data extraction?”

^c^ Item 3, “Was a comprehensive literature search performed?”

^d^ Item 4, “Was the status of publication (i.e. grey literature) used as an inclusion criterion?”

^e^ Item 5, “Was a list of studies (included and excluded) provided?”

^f^ Item 6, Were the characteristics of the included studies provided?

^g^ Item 7, Was the scientific quality of the included studies assessed and documented?

^h^ Item 8, Was the scientific quality of the included studies used appropriately in formulating conclusion?

^i^ Item 9, Were the methods used to combine the findings of studies appropriate?

^j^ Item 10, Was the likelihood of publication bias assessed?

^k^ Item 11, Was the conflict of interest stated?
