# Supplementary material for: Evidence for the effectiveness of anti-hypertensive medicines included on the Chinese National Reimbursement Drug List
Source: BMC Health Serv Res. 2019 Feb 11;19:112. doi: 10.1186/s12913-019-3937-0 (PMC6369556; doi:10.1186/s12913-019-3937-0)
Supplement: Supplementary file 2 — Table S2. Jadad score for the included randomized controlled trials. It provides information on whether certain included RCT had completed each of the 3 items, namely “randomization”, “double blinding” and “withdrawals and dropouts”, in the Jadad scale. (DOCX 21 kb) [file 12913_2019_3937_MOESM2_ESM.docx]

Additional file 2: **Table S2** Jadad score for the included randomized controlled trials.

|  | Dosage form | | RCT (First author, year) | Item 1: Randomization | Item 2: Double blinding | Item 3: Withdrawals and dropouts | Total score |
| --- | --- | --- | --- | --- | --- | --- | --- |
| **ACEIs** |  | |  |  |  |  |  |
| **Class B** |  | |  |  |  |  |  |
| Amlodipine benapril | oral release dosage form | | Cao J, 2012 (36) | 2 | 0 | 0 | 2 |
| Enalpril hydrochloro-thiazide | oral release dosage form | | Wang X, 2006 (48) | 2 | 1 | 1 | 4 |
| Imidapril | oral release dosage form | | Jiang X, 2005 (46) | 2 | 1 | 1 | 4 |
| Ramipril | oral release dosage form | | Tao B, 2006 (44) | 2 | 1 | 1 | 4 |
|  |  |  | Rokoss M J, 2005 (45) | 2 | 2 | 1 | 5 |
| Cilazapril | oral release dosage form | | Schiffrin EL, 2008 (49) | 2 | 1 | 1 | 4 |
| **β-blockers** |  | |  |  |  |  |  |
| **Class A** |  | |  |  |  |  |  |
| Metoprolol | injection | | Lu N, 2006 (37) | 1 | 1 | 0 | 2 |
| **Class B** |  | |  |  |  |  |  |
| Propranolol | | injection | Jiang X, 2001 (39) | 2 | 0 | 0 | 2 |
|  |  |  | Zuo W, 2007 (38) | 2 | 0 | 1 | 3 |
| **Calcium antagonist** |  | |  |  |  |  |  |
| **Class B** |  | |  |  |  |  |  |
| Diltiazem | injection | | Collabora-tive Group of Diltiazem, 2005 (47) | 2 | 1 | 1 | 4 |
| **Diuretics** |  | |  |  |  |  |  |
| **Class A** |  | |  |  |  |  |  |
| Furosemide | injection | | Huang G, 2008 (40) | 2 | 0 | 1 | 3 |
| **Class B** |  | |  |  |  |  |  |
| Torasemide | injection | | Zheng W, 2008 (41) | 1 | 1 | 1 | 3 |
| **Angiotensin Ⅱ receptor antagonist** |  | |  |  |  |  |  |
| **Class B** |  | |  |  |  |  |  |
| Olmesartan Medoxomil | oral release dosage form | | Liao Y, 2014 (43) | 2 | 0 | 1 | 3 |
| **Others** |  | |  |  |  |  |  |
| **Class B** |  | |  |  |  |  |  |
| Naftopidil | oral release dosage form | | Lu Q, 2000 (42) | 2 | 1 | 0 | 3 |
